# Mosquito community influences West Nile virus seroprevalence in wild birds: implications for the risk of spillover into human populations

**DOI:** 10.1038/s41598-018-20825-z

**Published:** 2018-02-08

**Authors:** Josué Martínez-de la Puente, Martina Ferraguti, Santiago Ruiz, David Roiz, Francisco Llorente, Elisa Pérez-Ramírez, Miguel Ángel Jiménez-Clavero, Ramón Soriguer, Jordi Figuerola

**Affiliations:** 1 Estación Biológica de Doñana (EBD-CSIC), Seville, Spain; 2 Servicio de Control de Mosquitos, Diputación de Huelva, Huelva, Spain; 3 Centro de Investigación en Sanidad Animal—Instituto Nacional de Investigación y Tecnología Agraria y Alimentaria (INIA-CISA), Valdeolmos, Madrid, Spain; 4 CIBER Epidemiología y Salud Pública (CIBERESP), Seville, Spain; 5 Present Address: UMR Mivegec, IRD, CNRS et Université de Montpellier, Montpellier, France

## Abstract

Mosquito community composition plays a central role in the transmission of zoonotic vector-borne pathogens. We evaluated how the mosquito community affects the seroprevalence of West Nile virus (WNV) in house sparrows along an urbanisation gradient in an area with the endemic circulation of this virus. We sampled 2544 birds and 340829 mosquitoes in 45 localities, analysed in 15 groups, each containing one urban, one rural and one natural area. WNV seroprevalence was evaluated using an epitope-blocking ELISA kit and a micro virus-neutralization test (VNT). The presence of WNV antibodies was confirmed in 1.96% and 0.67% of birds by ELISA and VNT, respectively. The VNT-seropositive birds were captured in rural and natural areas, but not in urban areas. Human population density was zero in all the localities where VNT-positive birds were captured, which potentially explains the low incidence of human WNV cases in the area. The prevalence of neutralizing antibodies against WNV was positively correlated with the abundance of the ornithophilic *Culex perexiguus* but negatively associated with the abundance of the mammophilic *Ochlerotatus caspius* and *Anopheles atroparvus*. These results suggest that the enzootic circulation of WNV in Spain occurs in areas with larger populations of *Cx. perexiguus* and low human population densities.

## Introduction

The mosquito-borne West Nile virus (WNV; *Flaviviridae*) circulates naturally in wild birds^1^. Occasionally, infected mosquitoes transmit WNV to mammals, which are dead-end hosts of this virus. Most WNV infections in humans are asymptomatic or associated with mild symptoms, and only a small percentage of patients develop more severe neurological diseases such as aseptic meningitis or encephalitis^2,3^. Nevertheless, in North America, where WNV was first detected in 1999 in New York City, WNV has spread throughout the country and caused hundred of human fatalities^2^. Contrary to the situation in North America, in Europe WNV infections are usually asymptomatic in birds^4–6^. WNV has been endemic in Spain since at least 2003, with a seroprevalence of up to 42.9% in some bird species^7–9^. However, only six cases of WNV disease in humans have ever been reported, one in 2004^10^, two in 2010^11^ and three in 2016^12^.

Mosquitoes of the genus *Culex* play a key role in WNV circulation in Europe^13,14^, although WNV has also been detected in mosquitoes belonging to the genera *Aedes*, *Anopheles* and *Culiseta*^14^. A multi-species Susceptible-Infectious-Recovered (SIR) transmission model published recently by Roche *et al*.^15^ suggests that an increase in vector species richness enhances pathogen transmission due to a concomitant greater abundance of vectors, which translates into more competent vectors for pathogen transmission^15^. In addition to the differences between mosquito capacity for transmitting WNV (vector competence), blood feeding patterns may determine the contact rate between mosquitoes and susceptible hosts and hence ultimately determine WNV epidemiology^16–18^. Muñoz *et al*.^17^ estimated the WNV transmission risk for different mosquito species in southern Spain based on mosquito abundance, vector competence and the fraction of blood meals taken from birds. These authors’ analysis indicated that *Cx. perexiguus* was the main vector for the enzootic cycle of WNV and that the risk of WNV transmission to humans was very low in the studied area^17^.

Here, we study the role of the abundance of mosquitoes and species richness explaining the seroprevalence of WNV in wild bird, the house sparrows (*Passer domesticus*), in southern Spain. Active circulation of WNV occurs here, as is shown by virus isolation from mosquitoes^14,19^, seroconversions in wild birds^20^, the presence of antibodies in juvenile birds^21^, and the incidence of disease in humans and horses^11,22^. In this region, house sparrows are common hosts of mosquitoes^17^, competent hosts for WNV^23^ and may play a key role in WNV amplification and transmission to humans^24–26^. Taking into account the above-mentioned studies, we first compared the prevalence of WNV antibodies in urban, rural and natural areas (defined in terms of human population density) to determine how the distributions of mosquitoes, WNV and people explain the low incidence of WNV in humans in Spain. Secondly, we tested the assumption in the Roche *et al*.^15^ model of a positive relationship between vector species richness and vector abundance, as well as the model prediction that vector richness should be positively related to WNV prevalence. Finally, we analysed the relationship between WNV seroprevalence and the abundance of mosquito species that, according to Muñoz *et al*.^17^, may contribute in different ways to WNV amplification.

## Results

In all, 340829 female mosquitoes belonging to 13 species and five genera were trapped. The commonest species was *Culex theileri* Theobald (n = 282891), followed in descending order by *Ochlerotatus caspius* Pallas (n = 21155), *Culex pipiens* Linnaeus (n = 19268), *Culex perexiguus* Theobald (n = 5939) and *Anopheles atroparvus* Van Thiel (n = 5387). In addition, 1237 females of the potential WNV vector *Culex modestus* Ficalbi were captured. The other species were trapped in relatively low numbers and for this reason—and also because they are not involved in the transmission of WNV—were not considered in any of the analyses (with the exception of the species richness calculation). A positive relationship was found between the overall abundance of mosquitoes and the richness of vector species (*est* = 2.45, *z* = 6.05, *p* < 0.001).

**Figure 1:**
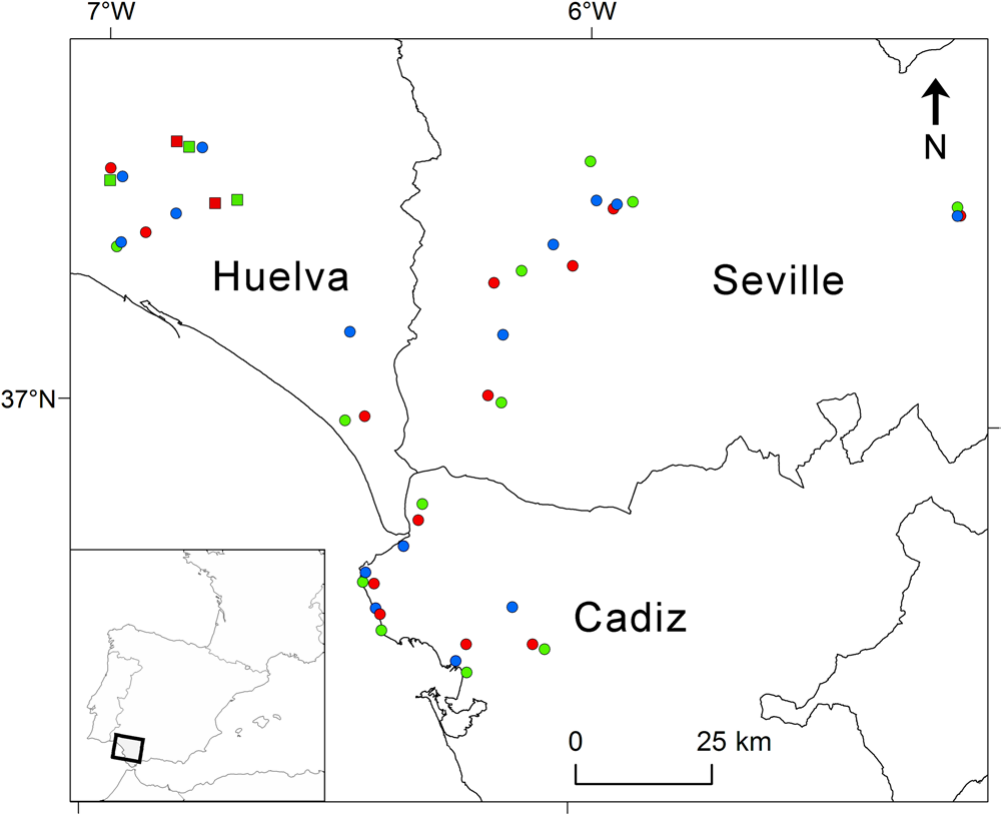
Distribution of the 45 studied localities that consisted of 15 urban (blue), 15 natural (green) and 15 rural (red) areas. Localities with birds with WNV positive sera according to VNT analyses are marked with squares. This map was created using ArcGIS v10.2.1 (ESRI, Redland).

Sera obtained from 2544 house sparrows were analysed to detect WNV antibodies. According to the ELISA tests, 50 birds (1.96%) from 18 different localities tested positively (Table 1), while 113 (4.44%) provided doubtful results. Of these birds, 17 (0.67% of the total individuals sampled) had neutralizing antibodies against WNV as confirmed by VNT (Table 1). These 17 WNV-positive birds were captured in five of the 45 studied localities, all of them in rural and natural areas in Huelva province (Fig. 1). WNV seroprevalence in these five localities ranged from 1.6% to 8.5%. Specific USUV-neutralizing antibodies were detected in a single bird (0.04%) captured in a natural area in Seville province. The human population density tended to be lower (0 in all cases) in areas with VNT-positive birds than in areas with negative cases (mean human population = 77.6, range: 0–1,424) (*est* = −1.90, *z* = 1.88, *p* = 0.06).

**Table 1:** Number of house sparrows sampled and number with WNV antibodies according to ELISA and VNT assays. Birds were captured in three habitat categories (natural, rural and urban areas) in three provinces (Cadiz, Huelva and Seville) in southern Spain.

	Birds sampled	ELISA positive	VNT positive
**Natural**			
Cadiz	154	3	0
Huelva	265	17	7
Seville	313	2	0
**Rural**			
Cadiz	225	0	0
Huelva	368	18	10
Seville	263	0	0
**Urban**			
Cadiz	238	4	0
Huelva	439	5	0
Seville	279	1	0
**Total**	2544	50	17

**Figure 2:**
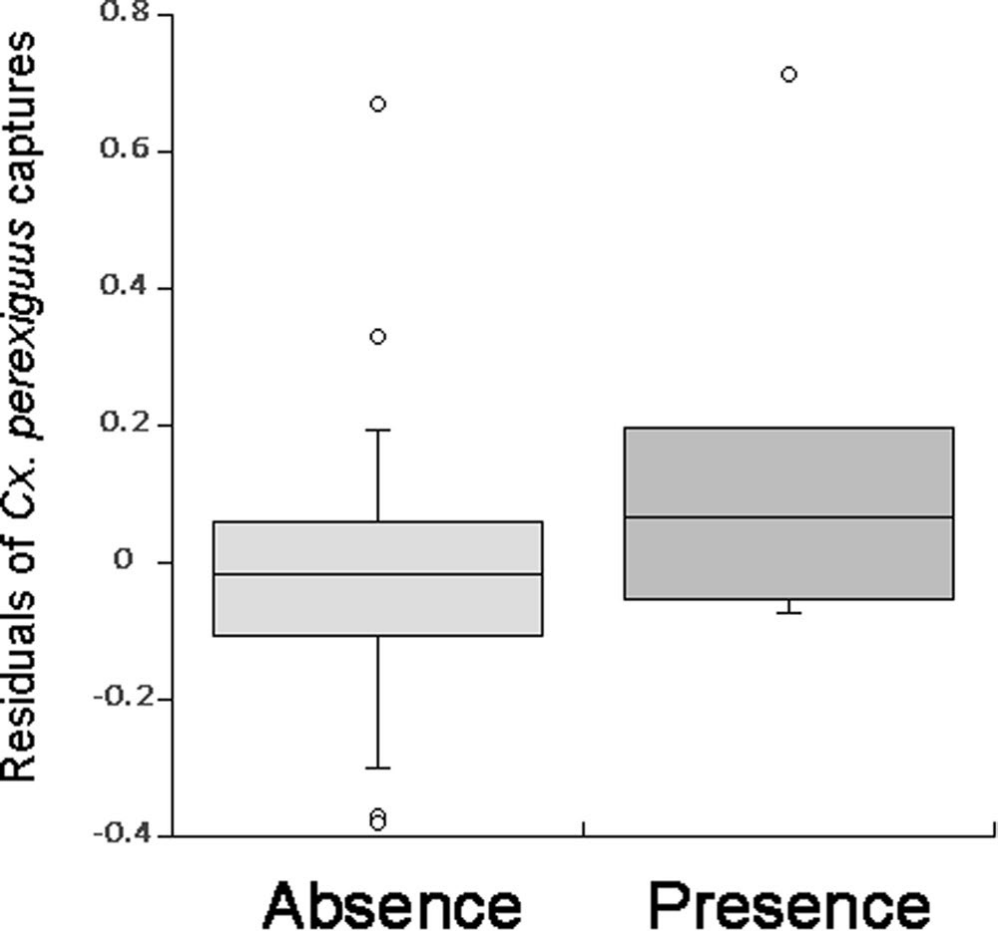
Number of *Culex perexiguus* captured in areas with and without WNV seropositive House sparrows, as determined by VNT.

The relationships between ELISA and VNT seroprevalence rates and the number of mosquitoes captured and species richness are summarised in Tables 2 and 3, respectively. Only those variables included in the selected models (those with ∆AIC ≤ 2 compared to the best model) are shown. WNV seroprevalences estimated by ELISA were positively related to mosquito richness and the number of *Cx. perexiguus* captured but negatively related to the number of *Oc. caspius* and *Cx. theileri* captured. Similarly, for the case of the model based on the WNV seroprevalence according to the VNT, the prevalence of neutralizing antibodies against WNV was positively related to the number of *Cx. perexiguus* captured (Fig. 2) but negatively associated with the number of both the *Oc. caspius* and *An. atroparvus*.

**Table 2:** Results of the LMMs explaining variance in WNV seroprevalence estimated by ELISA (N = 45 localities).

	*est*	*z*	*p*
Mosquito species richness	**2.24**	**2.18**	**0.03**
*Cx. perexiguus*	**1.93**	**2.04**	**0.03**
*Cx. theileri*	**−2.81**	**2.41**	**0.02**
*Oc. caspius*	**−2.54**	**2.14**	**0.02**
Explained variance	**35%**		

Only estimate (*est*), *z* and *p* values of the independent variables included in the final LMMs are shown; significant associations are marked in bold. Habitat category and the number of *Cx. pipiens* captured did not significantly improve the fit of the models.

**Table 3:** Results of the LMMs explaining variance in WNV seroprevalence estimated by micro virus-neutralization test (VNT) (N = 45 localities).

	*est*	*z*	*p*
Mosquito species richness	0.77	1.72	0.09
*Cx. modestus*	−0.69	1.64	0.10
*Cx. perexiguus*	**1.39**	**2.82**	**0.01**
*Cx. theileri*	−0.92	1.87	0.06
*Oc. caspius*	**−0.94**	**2.02**	**0.04**
*An. atroparvus*	**−1.01**	**1.99**	**0.05**
Explained variance	**44%**		

Only estimate (*est*), *z* and *p* values of the independent variables included in the final LMMs are shown; significant associations are marked in bold. Habitat category and the number of *Cx. pipiens* captured did not significantly improve the fit of the models.

## Discussion

Both West Nile virus and USUV antibodies were found in wild house sparrows from southern Spain. The seroprevalence of WNV in house sparrows estimated by VNT was positively related to the abundance of *Cx. perexiguus* but negatively to the abundances of both *An. atroparvus* and *Oc. caspius*. These results confirm the important role of *Cx. perexiguus* in the circulation of WNV in Spain. Transmission risk estimates based on abundances, vector competence and blood meal analyses indicate that the risk of transmission of WNV by *Cx. perexiguus* is at least an order of magnitude higher than for the other mosquito species analysed^17^. It is important to note that WNV has been detected in Spain mainly in *Cx. perexiguus* and *Cx. pipiens* pools^14,19^. Moreover, *Cx*. *perexiguus* is an abundant ornithophilic mosquito that commonly uses house sparrows as hosts^17,27,28^.

Interestingly, we found negative relationships between the abundance of two common mosquito species, *An. atroparvus* and *Oc. caspius*, and the prevalence of WNV antibodies in wild house sparrows. Both species have a mammal-biased feeding pattern, even though they can feed on birds^17,27^. Although WNV has been detected in wild collected *Oc. caspius*^29^, this species is described as an inefficient vector of WNV by the only experimental study of the vector competence of *Oc. caspius* conducted to date in Europe^13^. At least two factors help explain the negative association between these two mosquito species and WNV. Firstly, *Oc. caspius* prefers saltmarshes as larval breeding sites and *An. atroparvus* is commonest in sand dunes and scrubland, while *Cx. perexiguus* is frequently found in rice fields^30^. Consequently, *Oc. caspius* and *An. atroparvus* are probably more abundant in areas where *Cx. perexiguus* and/or other potential vector species for WNV such as *Cx. pipiens* and *Cx. modestus* are rarer. Secondly, the greater abundance of these mosquito species in the study area, where they feed mainly on mammals that are non-competent hosts for WNV, could lead to a reduction in the overall prevalence of WNV in birds. However, we were not able to identify any mechanisms that might support this hypothesis. Due to its mammal-biased diet and low vector competence, we would expect their abundance to have a low—but not negative—effect on WNV amplification. This is mainly because WNV transmission may be maintained by other vector-competent mosquito species present in the area.

In addition, we observed a positive association between mosquito species richness and the seroprevalence detected by ELISA. The same non-significant tendency was found for WNV neutralizing antibodies detected by VNT. ELISA is a less specific technique than VNT and, consequently, individuals with positive sera for ELISA but negative for VNT have probably been exposed to other unidentified flaviviruses antigenically related to WNV. Using a SIR model, Roche *et al*.^15^ concluded that mosquito species richness may increase the transmission success of vector-borne pathogens. However, such an association has never been tested empirically and could be the product of the assumption made in the model that species richness and vector abundance are positively related, a conjecture that, in fact, was supported by our data (see below). Consequently, our results support both the assumption of a positive relationship between vector richness and abundance, and the prediction of a positive relationship between vector richness and pathogen prevalence. Although *Cx. perexiguus* is the main vector of WNV in the area, other species such as *Cx. pipiens* and *Cx. modestus* may contribute significantly to WNV transmission^31,32^. These mosquito species, in addition to the others that co-exist in the area, could play a role in the transmission of certain flaviviruses. A number of flaviviruses have been isolated from mosquitoes (including *Cx. pipiens*) in Spain^19^, which potentially explains the positive correlation found between ELISA seroprevalence and mosquito species richness.

All positive cases of WNV-specific antibodies by VNT in bird sera were found in Huelva province, where evidence of WNV active circulation has existed since 2003, as demonstrated by the molecular detection of the virus in mosquitoes and the seroprevalence found in birds^33^. In addition, birds with WNV-specific antibodies by VNT were only detected in rural and natural habitats; none of the birds sampled in urban areas (n = 956) were seropositive. Moreover, the negative, marginally significant relationship we found between WNV seroprevalence and human population density may explain why WNV cases in humans are so uncommon in the study area despite the active circulation of the virus between vectors and avian hosts. Our results suggest that WNV, its main vector (*Cx. perexiguus*) and humans are not all present together in the same places. The seroprevalence of WNV in humans in southern Spain is very low (0.6%) and, mirroring the results for house sparrows in our study, a higher seroprevalence was detected in humans in rural areas than in suburban and urban areas^34^. Moreover, greater numbers of *Cx. perexiguus* were captured in natural and rural areas than in urban ones; likewise, the abundance of this species decreases as the percentage of land covered by built-up areas increases^35^. Indeed, only *Cx. pipiens* represents a risk for the transmission of WNV in urban areas^35^.

In conclusion, this study provides evidence of the central role of *Cx. perexiguus* in the enzootic circulation of WNV in southern Spain. The fact that WNV seropositive birds were found in both natural and rural areas, and tended to be present in areas with lower human densities, may explain the low incidence of WNV in humans in the area despite the local circulation of this virus between mosquitoes and wild birds.

## Materials and Methods

### Study area

This study was conducted in Andalusia, southern Spain (Fig. 1). This area is characterized by a Mediterranean climate with most precipitations concentrated during winter, while summer represents a long dry season. The study was conducted in 2013 at 45 different sites in Cadiz, Huelva and Seville provinces (southern Spain). The sampling sites (15 in each province) were situated in geographically close groups of three, each with one locality in a natural habitat, one in a rural habitat and one in an urban habitat (Fig. 1). The mean distance between localities within the same triplet was 5,740 m. Selection of the three habitat categories was performed after visual inspection of the areas based on the following criteria: urban habitats contained more densely human-populated areas than the other two habitat types; rural habitats had higher density of livestock than urban and natural areas; and natural habitats were selected on the basis of both lower human and livestock densities than in the other two habitat types, and a generally better conserved landscape.

### Mosquito and bird sampling and identification

Mosquitoes were captured at the 45 sampling sites in April–December, the period with maximum mosquito activity in southern Spain^30,36^. We used BG-sentinel traps baited with BG-lure and dry ice as a source of CO_2_, which is considered an effective method for mosquito diversity and abundance characterization^35^. At each site, once every 45 days, three traps were operated for 24 hours in each of the three localities of the same triplet. Overall, 135 traps (3 traps x 45 localities), with a mean distance between traps of 119 m (range 20–636 m), were employed during each mosquito trapping session for a total trapping effort of 810 trap nights. Mosquito sampling was conducted during days with favourable weather conditions (e.g. clear nights without rain). This procedure was repeated during 5–6 trapping sessions throughout the study period. Female mosquitoes were identified to species level following the morphological keys in Schaffner *et al*.^37^ and Becker *et al*.^38^. Mosquitoes belonging to the *univittatus* complex were identified as *Culex perexiguus* based on male genitalia (see Harbach^39^). For the case of samples compromising several thousands of mosquitoes captured per trap per night, we visually identified 500 individuals. These 500 mosquitoes were separated in five groups of 100 individuals, which were weighted to the nearest 0.001 g. This approach was used to estimate the proportion of individuals of each species for the rest of the sample based on the weight of the total number of mosquitoes captured^35^. Mosquito species richness, which ranged from 2 to 10, was calculated as the number of different species captured at each locality during the sampling period^35^. For each locality, the mean number of captures of the five commonest mosquito species in the study area (*Anopheles atroparvus*, *Ochlerotatus caspius*, *Culex theileri*, *Culex pipiens* and *Cx. perexiguus*) and of *Cx. modestus*, a potential WNV vector in the area^14,17,31^, were calculated.

House sparrows were sampled using mist-nests at the same localities during capture sessions in July–October, i.e. immediately after the breeding season to maximize the capture of juvenile birds and to better reflect virus circulation during the season from hatching until capture. Each bird was individually marked with a metal ring, sexed and aged^40^. A blood sample was taken from the jugular vein of each bird using a sterile syringe and preserved in a cool-box during the fieldwork session. In the laboratory, blood was allowed to clot at 4 °C overnight and was then centrifuged for 10 minutes at 4,000 rpm to separate the serum from the cellular fractions. Serum samples were frozen at −80 °C until further analysis.

### WNV antibodies detection

Serum samples from birds were analysed with the epitope-blocking ELISA Kit Ingezim West Nile Compac (INGENASA, Madrid, Spain) to determine the presence of WNV antibodies^41^. Positive results from ELISA may reflect past infections by WNV or even other unidentified flaviviruses circulating in the area. The cut-off value of this commercial ELISA test is set at 30% percentage of inhibition, while samples showing a percentage of inhibition between 30% and 40% are considered doubtful samples as established by the manufacturer^41^. All samples producing ELISA positive and/or doubtful results were subsequently analysed by a comparative micro virus-neutralization test (VNT) using WNV (strain Eg-101) and Usutu virus (USUV; strain SAAR1776) since the circulation of these flaviviruses has been demonstrated in the study area^42^. This confirmatory test allow to differentiate the specific antibodies against WNV from those elicited by other related flaviviruses. Neutralization titres were assigned based on the highest dilution of each serum capable of neutralizing the infection *in vitro*. Separate VNT were performed using serial (two-fold) dilutions (1:10–1:1280) of each serum sample using a micro-VNT method^21^. For a given sample, WNV-specific antibody responses were assigned when the observed VNT titres against WNV were at least four times higher than those observed against USUV^43^.

### Human density quantification

We estimated the density of human population in the studied areas as the number of people living in a grid of 250 × 250 m. This information was obtained from the Andalusian Institute of Statistics and Cartography based on the number of residents registered in the local population census on 1 January 2013 (Base de Datos Longitudinal de Población de Andalucía). This variable was log-transformed to normalize its distribution.

### Statistical analyses

To estimate WNV seroprevalence we controlled for variables that operate at individual level (i.e. age, sex and date of capture) and others that operate at locality level (i.e. mosquito species richness and abundance of the different mosquito species). For this reason two-step analyses were performed. First, we fitted a generalized linear model to the seroprevalence of WNV using binomial distributed errors and including bird sex (fixed factor: male or female), age (fixed factor: juvenile or adult), month (continuous variable) and locality (fixed factor) as independent variables. Two different models were fitted using the results of ELISA and VNT as the dependent variable, respectively. Second, least square means (*lsmeans*) were calculated by retaining bird age and sampling locality, the only two significant factors explaining variance between individuals in WNV seroprevalence according to the previous models. This procedure allowed us to calculate both the ELISA and VNT seroprevalences for each of the 45 localities while controlling for the potential confounding effect of bird age. Third, two Linear Mixed-effects Models (LMM) were fitted using the lsmeans for ELISA and VNT seroprevalences as dependent variables. ‘Province’ and ‘triplets’ were included as random factors to account for the geographical stratification of the sampling design, and models were fitted using maximum likelihood and normal distributed errors. The independent variables included in these models were the three habitat categories (fixed factor: urban, rural or natural), the number of captures of each of the six main mosquito species found and species richness (continuous variables). Variance Inflation Factors (VIF) were checked to exclude collinearity between independent variables^44^ and Akaike’s Information Criterion (AIC) was used to select the best final models for each ELISA and VNT LMM model. Parameters were estimated by model averaging of all models with ∆AIC ≤ 2^45^, which were considered to similarly support the data. To normalize their distribution, the numbers of each mosquito species captured were log-transformed and the distribution of all predictors and model residuals were checked using *qq plots* in R software. We calculated the respective marginal coefficient of determination (R^2^) for the fixed and random effects of the models according to Nakagawa & Schielzeth^46^.

Finally, two additional LMMs were fitted. One to test the model assumption of Roche *et al*.^15^ of a positive correlation between mosquito richness and total abundance, and the other to compare the density of human population, as measured in Ferraguti *et al*.^35^, at sampling sites with and without VNT positive birds. All the statistical analyses were conducted in R (v. 2.14.2; R Development Core Team 2005) using the packages *vegan*, *lme4*, *car*, *arm*, *Matrix*, *Rcpp*, *MASS*, *MuMIn* and *lsmeans*.

### Ethics statement

Bird sampling and mosquito trapping were performed with the necessary permits issued by the regional Department of the Environment (Consejería de Medio Ambiente, Junta de Andalucía) and in accordance with relevant guidelines and regulations. Procedures were approved by the Ethical Committee of CSIC and complied with current Spanish laws. Surveys and sampling on private land and in private residential areas were conducted with all the necessary permits and consent, and in the presence of owners. This study did not affect any endangered species.
